# *In vitro* up-regulation of expression of GITR on human CD8+ cells

**DOI:** 10.4103/0971-6866.55214

**Published:** 2009

**Authors:** Yasmin Thanavala

**Affiliations:** Department of Immunology, Roswell Park Cancer Institute, Buffalo, NY, 14263, USA

Glucocorticoid-induced TNFR-related protein (GITR also known as TNFRSF18), a member of the tumor necrosis factor superfamily (TNFSF), is expressed on many cells of the innate and adaptive immune system including NK cells, PMN, monocytes, macrophages, B cells and mast cells. As with other members of the TNFR family, signaling through GITR can induce cell death or survival.[1] The natural ligand of GITR (GITRL) is a type 11 trans-membrane protein expressed on endothelial cells, macrophages, dendritic cells and B cells but is absent on T cells.[2,3] The GITRL expression is increased by pro-inflammatory stimuli. GITR is expressed at different levels on CD4+ and CD8+ cells and GITR levels have been shown to be up-regulated after T cell activation. GITR is expressed constitutively at high levels on CD4+CD25+ T reg cells. The GITR-GITRL interactions have been shown to reverse suppression mediated by T regulatory cells. Most of these studies have utilized a GITR specific monoclonal antibody called DTA1. While several studies have shown that DTA1 does not lead to Treg cell depletion other studies do report 30-50% depletion of CD4+CD25+ T cells following DTA1 treatment. It has been difficult to decide if the cause of augmentation of immune responses following administration of DTA1 is stimulation of responder T cells, depletion of T reg cells or a combination of both. A further wrinkle in the above scenarios is that DTA1 treatment may also deplete activated T cells that express high levels of GITR. Long term treatment of infant mice with GITR specific agonistic antibodies has resulted in the development of auto-immune gastritis. Similarly, treatment of mice during the development phase of EAE resulted in more severe disease, exacerbates collagen-induced arthritis and allergic airway inflammation. In the tumor setting, mice treated with GITR specific antibodies on the day of B16 melanoma inoculation and for the next seven days showed potent immunity.[4‐6] Moreover, other studies using methylcholanthrene induced tumor cells showed that GITR specific antibodies could be given after tumor injection and still showed generation of anti-tumor immunity. The jury appears to still be out on the normal physiological role of GITR *in vivo*. Most of the studies thus far have been done on mice where effects on both CD4+ and CD8+ cells have been documented. Relatively less work has been done using human PBLs though, of course, in the original paper by Gurney *et al*. when they identified the human ortholog of mouse GITR they did study GITR mRNA expression on human PBL and showed that the levels were up-regulated following treatment with anti-CD3 antibody. However, purified T cell subsets have not been analyzed. The manuscript by Chattopadhyay and Chakraborty, in this issue, describes the *in vitro* up-regulation of expression of GITR on human CD8+ cells purified from PBLs of three donors when stimulated with CD3 and points out that GITR levels were down-regulated when the CD8+ cells were treated with a JNK specific inhibitor but not inhibitors of other kinases.
